# Sinomenine Provides Neuroprotection in Model of Traumatic Brain Injury via the Nrf2–ARE Pathway

**DOI:** 10.3389/fnins.2016.00580

**Published:** 2016-12-23

**Authors:** Youqing Yang, Handong Wang, Liwen Li, Xiang Li, Qiang Wang, Hui Ding, Xiaoliang Wang, Zhennan Ye, Lingyun Wu, Xiangsheng Zhang, Mengliang Zhou, Hao Pan

**Affiliations:** ^1^Department of Neurosurgery, Jinling Hospital, Clinical Medical College of Southern Medical University (Guangzhou)Nanjing, China; ^2^Department of Neurosurgery, Jinling Hospital, School of Medicine, Nanjing UniversityNanjing, China

**Keywords:** traumatic brain injury, nuclear factor erythroid 2-related factor 2, sinomenine, neuroprotection, reactive oxygen species

## Abstract

The neuroprotective effect of sinomenine (SIN) has been demonstrated in several brain injury models. However, its role and molecular mechanism in traumatic brain injury (TBI) remain unknown. In this study, we investigated the neuroprotective effects of SIN in the weight-drop model of TBI in male ICR mice. Mice were randomly divided into the sham and TBI groups, SIN (10 mg/kg, 30 mg/kg and 50 mg/kg, administered intraperitoneally) or equal volume of vehicle was given at 30 min after TBI. Treatment with 30 mg/kg SIN significantly improved motor performance and alleviated cerebral edema. However, treatment with 10 mg/kg or 50 mg/kg SIN did not exhibit a better outcome. Therefore, we chose 30 mg/kg SIN for our subsequent experiments. SIN significantly increased the expression of Bcl-2 and decreased that of cleaved caspase-3, indicating that SIN is anti-apoptotic. This was confirmed by the observation that SIN-treated animals had fewer apoptotic neurons. Cortical malondialdehyde content, glutathione peroxidase (GPx) activity and superoxide dismutase (SOD) activity were restored in the group that received SIN. Furthermore, Western blot and immunofluorescence experiments showed that SIN enhanced the translocation of nuclear factor erythroid 2-related factor 2 (Nrf2) to the nucleus. SIN administration also significantly upregulated the expression of the downstream factors heme oxygenase 1 and NAD(P)H:quinone oxidoreductase 1 at pre- and post-transcriptional levels. Together, these data demonstrate that SIN exerts a neuroprotective effect in a model of TBI, possibly by activating the Nrf2–antioxidant response element (ARE) pathway.

## Introduction

Traumatic brain injury (TBI) remains a major public health problem in modern society, leading to high medical costs, morbidity and mortality (Zipper and Mulcahy, 2003; Tsai et al., 2013). The pathological process of TBI involves primary and secondary injury. After a primary brain insult, a complex series of endogenous events are triggered, including oxidative stress, glutamate excitotoxicity, activation of the inflammatory response, loss of ionic homeostasis, and increased vascular permeability (Werner and Engelhard, 2007; Bell et al., 2009; Cornelius et al., 2013), leading to further neuronal degeneration and apoptosis. These subsequent pathological events are referred to as secondary brain injury. Despite the efforts focused on seeking effective ways to alleviate the secondary injury, to date, most approaches to the treatment of TBI targeting a single injury mechanism have failed in clinical trials (Sun et al., 2015).

Oxidative stress plays an important role in secondary injury (Bains and Hall, 2012). It occurs when the production of reactive oxygen species (ROS) exceeds the cell ability to detoxify. The excessive production of ROS damages cellular components including lipids, proteins, and DNA, leading to a decline in physiological function and cell death (Ansari et al., 2008; Adibhatla and Hatcher, 2010).

The transcription factor nuclear factor erythroid 2-related factor 2 (Nrf2) is a key protein in the reduction of oxidative stress (Yan et al., 2008). Under normal conditions, kelch ECH associating protein 1 (KEAP1), a cytosolic repressor protein that binds to Nrf2, retains Nrf2 in the cytosol and facilitates its proteasomal degradation. Once the cell encounters stimulations such as oxidative stress, Nrf2 dissociates from KEAP1 and translocates into the nucleus (de Vries et al., 2008). By binding to the antioxidant response element (ARE), Nrf2 induces the production of a battery of endogenous enzymes, such as superoxide dismutase (SOD), glutathione peroxidase (GPx), nicotinamide adenine dinucleotide phosphate, quinine oxidoreductase-1 (NQO1), and heme oxygenase-1 (HO-1). Together, these free radical scavenging enzymes represent a powerful antioxidant defense mechanism (de Vries et al., 2008; Ma, 2013). The Nrf2-ARE signaling pathway is activated in several central nervous system (CNS) diseases, including TBI (Wang et al., 2007; Chen et al., 2011), and is considered a protective molecular mechanism against TBI (Yan et al., 2009).

Sinomenine (7,8-didehydro-4-hydroxy3,7-dimethoxy-17-methyl-9α, 13α, 14α-morphinan-6-one; SIN) is an active alkaloid isolated from the Chinese medicinal herb *Sinomeniumacutum*, and is used in China for the clinical treatment of rheumatoid arthritis and mesangial proliferative nephritis (Xu et al., 2008; Cheng et al., 2013). It has a variety of pharmacological properties including immunosuppression, anti-inflammation and cytoprotection (Qian et al., 2007; Cheng et al., 2009; Wang and Li, 2011). SIN exerts neuroprotection in several CNS disease models, including cerebral ischemia (Wu et al., 2011), intracerebral hemorrhage (Yang et al., 2014), and neurodegenerative diseases (Qian et al., 2007). However, few studies have addressed the neuroprotective effect of SIN in TBI. Therefore, the purpose of the present study was to determine whether SIN administration after TBI could attenuate brain injury in a TBI model.

## Materials and methods

### Animals

Male ICR mice weighing 28–32 g were obtained from the Animal Center of Jinling Hospital. The animals were housed in a controlled environment with a reversed 12/12 h light/dark cycle and free access to food and water and were acclimatized for at least 4 days before any experiment. All procedures involving animals were approved by the Animal Care and Use Committee of Southern Medical University, Guangzhou, China, and conformed to the National Institutes of Health Guide for the Care and Use of Laboratory Animals.

### Model of TBI

The model of TBI used in the present study was based on Marmarou's weight-drop model with some modifications as previously described by Flierl et al. (Flierl et al., 2009; Zhang et al., 2016). Mice were anesthetized in a closed container on a wire mesh over ether-soaked cotton and then placed onto the platform directly under the weight of the weight-drop device.

The impact area (left anterior frontal region, 1.5 mm lateral to the midline on the midcoronal plane) was located, and a 200 g weight was released and dropped onto the skull from a height of 2.5 cm. Mortality rate from apnea was reduced by early respiratory support. The scalp wound was closed with standard suture material. After recovering from anesthesia, mice were returned to their cages with food and water provided *ad libitum*. Sham-injured animals underwent the same procedure except for the weight drop.

A total of 214 mice were used in our experiment. No animals died in the sham-injured group and sham + SIN group. Ten (TBI, *n* = 3; TBI + vehicle, *n* = 3; TBI + 10 mg/kg SIN, *n* = 1; TBI + 30 mg/kg SIN, *n* = 2; TBI + 50 mg/kg SIN, *n* = 1) TBI mice died before sacrifice, and the mortality rate was 6.7%.

### Experimental groups and drug treatment

Animals were randomly assigned to the following groups: (1) sham (*n* = 42); (2) sham + SIN (50 mg/kg i.p.) (*n* = 12); (3) TBI (*n* = 42); (4) TBI + vehicle (*n* = 42); (5) TBI + SIN (10 mg/kg i.p.) (*n* = 12); (6) TBI + SIN (30 mg/kg i.p.) (*n* = 42) and (7) TBI + SIN (50 mg/kg i.p.) (*n* = 12). SIN was purchased from Sigma-Aldrich (St. Louis, MO) and freshly prepared in saline containing 1% dimethylsulfoxide (DMSO) just before injection. Animals in the TBI + SIN and TBI + vehicle groups received SIN or equal volumes of 1% DMSO, respectively, 30 min after TBI. Animals in the sham + SIN and sham + vehicle groups received SIN or vehicle intraperitoneally 30 min after surgery. The mice were sacrificed 24 h after TBI for biochemical and histological analyses. The doses used in this study were based on a study of neuroprotection of SIN in a middle cerebral artery occlusion model (Wu et al., 2011).

### Neurological evaluation

Neurological function was evaluated by the grip test which was developed on the basis of the test of gross vestibulomotor function as described elsewhere (Bermpohl et al., 2006; Xu et al., 2014). Briefly, mice were placed on a thin, horizontal, metal wire (45 cm long) that was suspended between two vertical poles 45 cm above a foam pad and were allowed to traverse the wire for 60 s. The latency that a mouse remained on the wire within a 60-s interval was measured, and wire grip scores were quantitated using a 5-point scale (Table 1). The grip test was performed in triplicate, and a total value was calculated for each mouse. The test was performed by an investigator who was blinded to experimental grouping.

**Table 1:** **Behavior scores**.

**Behavior**	**Points**
Unable to remain on the wire for less than 30 s	0
Failed to hold on to the wire with both fore paws and hind paws together	1
Held on to the wire with both fore paws and hind paws but not the tail	2
Used its tail along with both fore paws and both hind paws	3
Moved along the wire on all four paws plus tail	4
Scored four points also ambulated down one of the posts used to support the wire	5

### Brain water content

Brain water content was measured according to a previous study (Xu et al., 2013). In brief, the brain was removed and placed on a cooled brain matrix 24 h after TBI. The brainstem and cerebellum were removed, and the remaining ipsilateral tissue was weighed immediately to obtain the wet weight (ww). Then the hemisphere was dried at 80°C for 72 h and weighed to obtain the dry weight (dw). We calculated water content as a percentage using the following formula: (ww − dw)/ww × 100%.

### Tissue processing

For Western blot and real-time quantitative polymerase chain reaction (RT-PCR) analysis, animals were anesthetized with chloral hydrate 24 h after TBI and perfused through the left cardiac ventricle with 0.9% normal saline solution at 4°C. Ipsilateral cerebral cortex tissue 3 mm from the margin of the contusion site was dissected on ice, immediately frozen in liquid nitrogen, and stored at −80°C until use. For immunofluorescence staining and terminal deoxynucleotidyl transferase-mediated biotinylated deoxyuridine triphosphate nickend labeling (TUNEL), after being deeply anesthetized with chloral hydrate 24 h after TBI, animals were perfused with 0.9% normal saline solution followed by 4% buffered paraformaldehyde, and the brains were immersed in 4% buffered paraformaldehyde overnight (all at 4°C).

### Determination of malondialdehyde (MDA), superoxide dismutase (SOD), and glutathione peroxidase (GPx)

Tissue samples were homogenized in 2 ml of phosphate buffer (10 mM; pH 7.4). After centrifugation at 12,000 rpm for 15 min at 4°C, MDA, SOD, and GPx content in the supernatant was measured using a spectrophotometer (Nanjing Jiancheng Biochemistry Co., Nanjing, China) according to the manufacturer's instructions. Protein concentrations were determined using the Bradford method. MDA content was expressed as nmol/mg protein, and SOD and GPx activity were expressed as U/mg protein.

### Tunel analysis

Apoptosis was determined using TUNEL, and included analysis of DNA fragmentation assays based on 3H-thymidine and 5-bromo-2-deoxyuridine (In Situ Cell Death Detection Kit, TMR red; Sigma-Aldrich), according to the manufacturer's instructions. The slides were then washed with PBST three times for 30 min prior counterstaining with 4′,6-diamidino-2-phenylindole (DAPI) for 15 min. After three more washes, the slides were coverslipped with anti-fade mounting medium for further study. The TUNEL-positive cells were counted by an observer who was blind to the experimental groups. To evaluate the extent of cell apoptosis, the apoptotic index was defined as the average number of TUNEL-positive cells in each section counted in six microscopic fields.

### Total/nuclear protein extraction and western blot analysis

Proteins were extracted using the Nuclear and Cytoplasmic Protein Extraction Kit (Beyotime Biotech Inc., Nantong, China) according to the kit instructions, and protein concentrations were determined using the Bradford method. Equal amounts of protein per lane (50 μg) were separated by 10 or 12% sodium dodecyl sulfate-polyacrylamide gel electrophoresis and transferred to polyvinylidene-difluoride membranes (Millipore, Bedford, MA, USA). The membranes were incubated in blocking buffer (Tris buffered saline/0.05% Tween 20 [TBST] containing 5% skim milk) for 2 h at room temperature, then overnight at 4°C with primary antibodies (all raised in rabbit), as follows: anti-Nrf2, anti-HO-1 and anti-NQO-1 (1:1000, all from Abcam, Cambridge, MA, USA), anti-Bcl-2 (1:200, Santa Cruz Biotechnology, Santa Cruz, CA, USA), anti-cleaved caspase-3 and anti-Histone 3 (1:1000, Cell Signaling Technology, Beverly, MA, USA), anti-β-actin (1:5000, Bioworld Technology, St. Louis Park, MN, USA). After three 10 min washes with TBST, the membranes were incubated with goat anti-rabbit horseradish peroxidase (HRP)-conjugated IgG (1:5000, Bioworld Technology) for 2 h at room temperature. Protein bands were visualized by enhanced chemiluminescence western blot detection reagents (Millipore) and quantification was performed by optical density methods using ImageJ software (NIH). Proteins of interest were normalized to β-actin or histone 3.

### qRT-PCR

Total RNA was extracted from the ipsilateral cortex with RNAiso Plus (Takara Bio, Dalian, China). The concentration and purity of total RNA were determined with a spectrophotometer (OD260/280 1.8–2.0) and 1% agarose gel electrophoresis. To avoid RNA degradation, some of the RNA was immediately reverse-transcribed to cDNA using the PrimeScript RT reagent kit (Takara Bio); surplus RNA was kept at 80 °C. The primers were designed according to PubMed GenBank and synthesized by Invitrogen Life Technologies (Shanghai, China). The sequences were as follows: NQO1: F, 5′-CATTCTGAAAGGCTGGTTTGA-3′; R, 5′-wordCTAGCTTTGATCTGGTT-GTCAG-3′; OH-1: F, 5′-wordATCGTGCTCGCATGAACACT-3′; R, 5′-wordCCAACACTGC-ATTTACATGGC-3′; β-actin: F, 5′-wordAGTGTGACG-TTGACATCCGTA-3′; R, 5′-wordGC-CAGAGCAGTAATCTCCTTCT-3′. qRT-PCR analysis was performed using the Mx3000P System (Stratagene, San Diego, CA, USA), applying real-time SYBR Green PCR technology. All samples were analyzed in triplicate. β-actin was used as an endogenous reference “housekeeping” gene.

### Immunofluorescence for Nrf2

Cryostat frozen sections (8 μm thick) were mounted on gelatin-coated slides, which were warmed at room temperature for 30 min. Slides were washed three times in PBS for 10 min each time before immunofluorescence staining. Based on the established immunostaining protocol, slides were incubated in blocking buffer (10% normal goat serum in PBS containing 0.1% Triton X-100) for 2 h followed by overnight incubation at 4°C with rabbit anti-Nrf2 (1:100, Abcam) and anti-NeuN (1:100, Millipore). The next day, after three more 5 min washes in PBS, the slides were incubated with appropriate secondary antibodies (Alexa Fluor 488, 1:200) for 2 h at room temperature. The slides were washed three times in PBS, counterstained with DAPI for 2 min, rinsed with PBS, and coverslipped with mounting medium. Fluorescence microscopy imaging was performed using a Zeiss HB050 inverted microscope system and handled by Image-Pro Plus 6.0 software (Media Cybernetics, USA) and Adobe Photoshop CS5 (Adobe Systems, USA). The specificity of the immunofluorescence reaction was confirmed using a negative control in which the primary antibody was replaced with PBS. Six random fields of vision (200 ×) were chosen for each coronal section. Four sections from each animal were used for quantification. The final average number of positive cell in the four sections was used as the data for each sample. Data are presented as the mean fluorescence intensity per 200 × magnification field. The entire process was conducted by two pathologists blinded to the grouping.

### Statistical analysis

All data used for statistical analysis are expressed as the mean ± SEM. One-way ANOVA and Tukey's test were used to analyze differences between groups except for the neurobehavioral scores, which were analyzed using nonparametric tests (Kruskal–Wallis followed by Dunn's test). SPSS 20.0 was used for statistical analysis (IBM Corp., Armonk, NY, USA). Statistical significance was inferred at *P* < 0.05.

## Results

### SIN improves recovery of motor performance and alleviates cerebral edema after TBI

**Figure 1:**
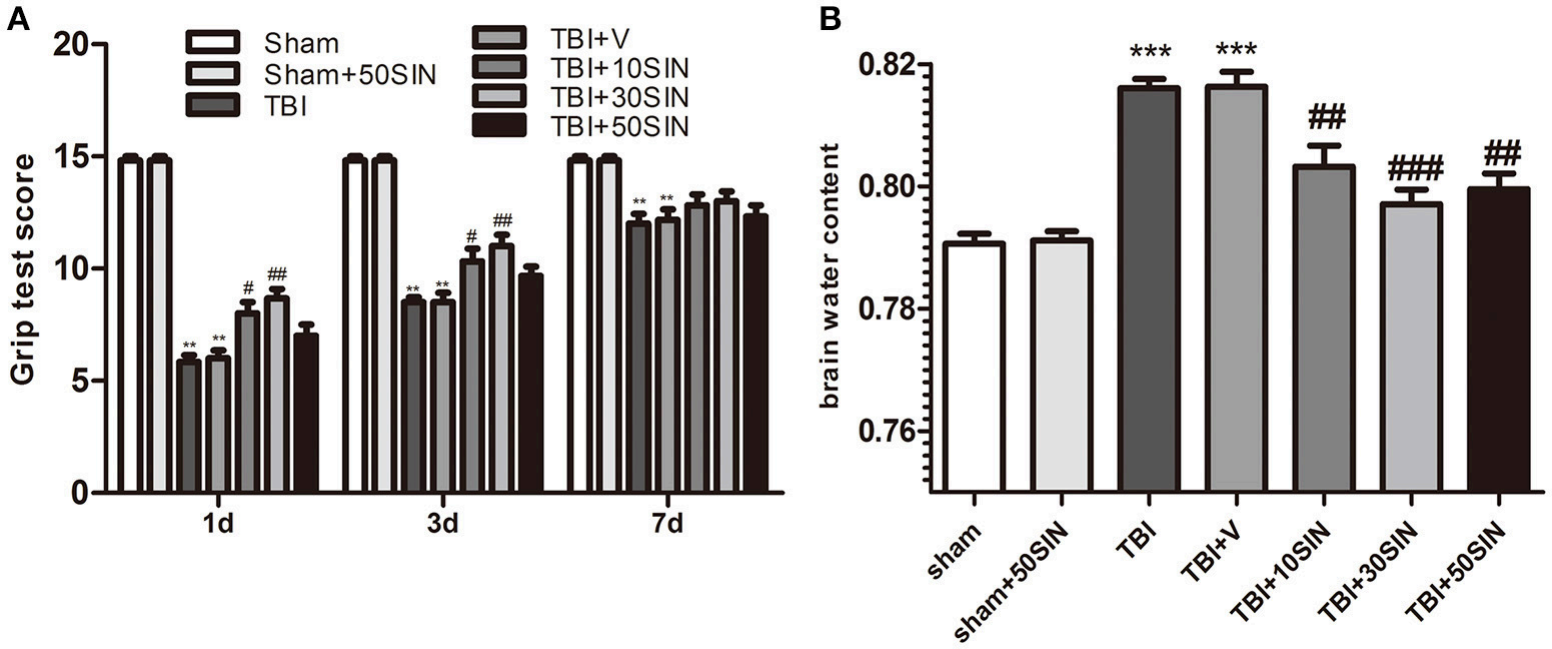
**SIN protects against secondary brain injury in a mice model of TBI**. Mice were subjected to TBI and then received 10, 30, and 50 mg/kg of SIN or vehicle (dimethyl sulfoxide) 30 min later. Grip test score were examined at 1 day, 3 days, and 7 days after TBI while brain water content were evaluated at 1 day after TBI. **(A)** Within 3 days after TBI, performance of the 10 and 30 mg/kg SIN-treated groups was significantly better than in the vehicle group; however, there was no significant difference between the 50 mg/kg group and the vehicle group. This effect was no longer significant 7 days after TBI between all groups treated with different doses of SIN and the vehicle-treated group. *n* = 6 per group. **(B)** Brain water content was significantly greater in the TBI and TBI + vehicle groups than in the sham or sham + SIN groups. SIN significantly attenuated brain water content compared with the TBI+vehicle group, with no significant difference between doses. *n* = 6 per group. Data are presented as mean ± SEM. ^**^P < 0.01 vs. sham; ^***^P < 0.001 vs. sham; ^#^*P* < 0.05, ^##^*P* < 0.01, ^###^*P* < 0.001 vs. TBI + vehicle group. TBI, qtraumatic brain injury; SIN, sinomenine.

To examine whether SIN provides neuroprotection after TBI, animals were grouped as follows: (1) sham; (2) sham + SIN (50 mg/kg); (3) TBI; (4) TBI + vehicle; (5) TBI + SIN (10 mg/kg); (6) TBI + SIN (30 mg/kg) and (7) TBI + SIN (50 mg/kg). Motor performance was evaluated using the grip test 1 day, 3 and 7 days after TBI. All animals were trained on the task 24 h before TBI. The sham group showed no difference between different time points and there was no difference between the TBI group and the vehicle-treated group. All groups exhibited an improved motor performance over time after TBI. Within 3 days after TBI, the performance of the groups that received SIN was significantly better than those that received vehicle. Larger doses such as 50 mg/kg, however, did not exhibit a better outcome than 30 mg/kg (*P* > 0.05; Figure 1A).

We then measured brain water content to confirm the neuroprotective effect of SIN. Brain water content after TBI was markedly lower in all three SIN-treated groups than in animals that received vehicle (*P* < 0.01, *P* < 0.001, and *P* < 0.01, for 10, 30, and 50 mg/kg, respectively; Figure 1B). Consistent with the grip test, the 30 mg/kg dose had a slightly, but not significantly, greater effect in reducing TBI-induced brain edema than the other doses (*P* > 0.05; Figure 1B). These data confirm that SIN is neuroprotective against TBI, 30 mg/kg provided better recovery when comparing the results of these two tests, maybe in further tests other doses would have different dynamics. Therefore, we used this dose in the subsequent studies.

### Sinomenine administration attenuated neuronal apoptosis in the brain after TBI

**Figure 2:**
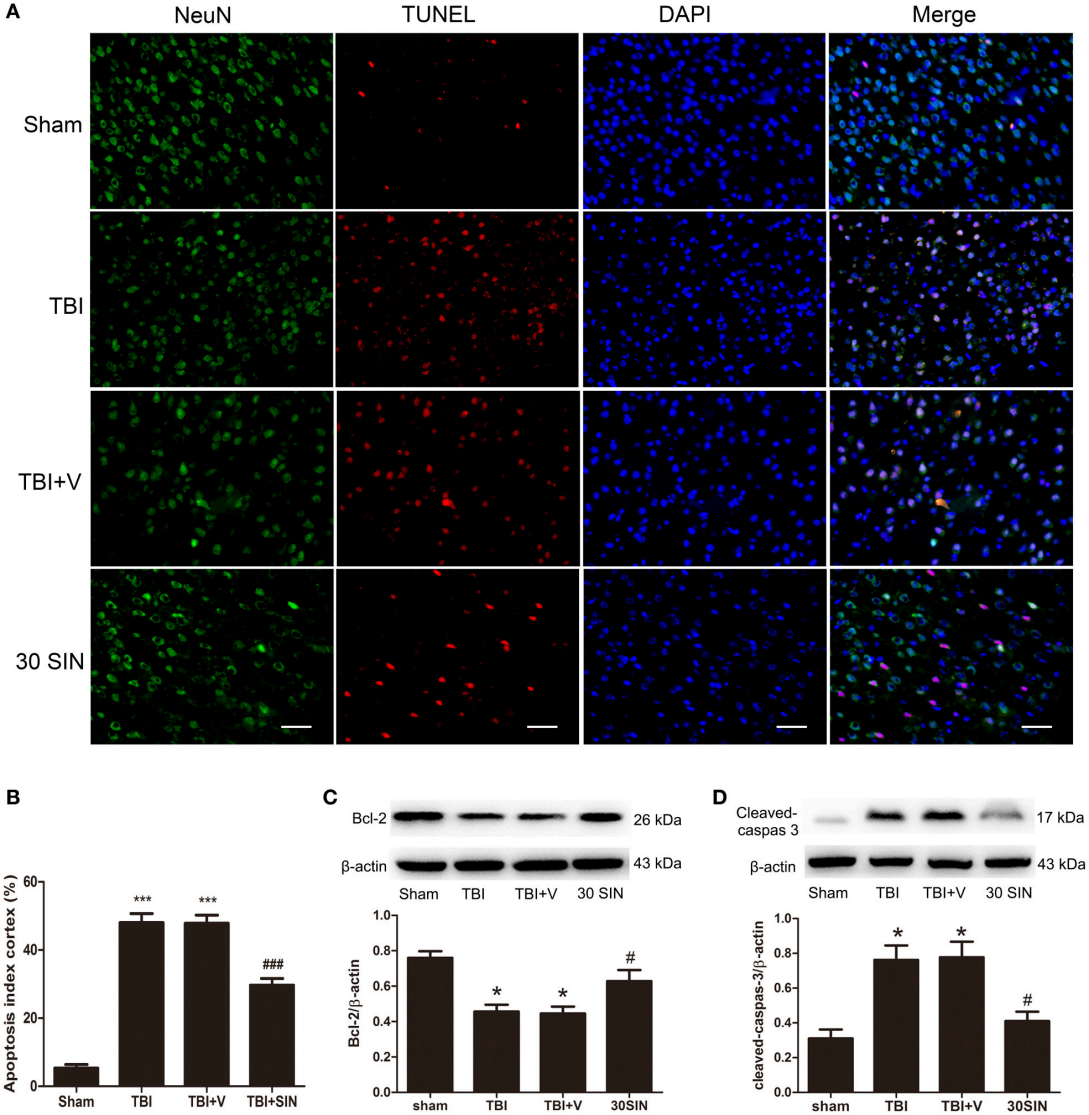
**SIN treatment attenuated TBI-induced neuronal apoptosis 24 h after TBI. (A)** Representative TUNEL staining of brain sections of sham, TBI, vehicle-treated and SIN-treated mice at 24 h post-injury. **(B)** SIN treatment significantly decreased the number of TUNEL-positive neurons after TBI. Quantification showed that SIN-treated mice had significantly fewer TUNEL-positive neurons than the vehicle-treated mice. Representative immunoblots showing the protein levels of Bcl-2 **(C)** and cleaved caspase-3 **(D)** in the sham, TBI, TBI+vehicle, and TBI+SIN groups at 24 h after TBI. *n* = 6 per group. Data are presented as mean ± SEM. ^*^P < 0.05 and ^***^P < 0.001 vs. sham group, ^#^*P* < 0.05, and ^###^*P* < 0.001 vs. TBI + vehicle group.

To determine whether the neuroprotective effects of SIN can be detected at a histopathological level, NeuN/TUNEL double immunofluorescence staining was performed to evaluate neuronal apoptosis. The total number of TUNEL and NeuN double-stained cells was significantly greater in the TBI and TBI + vehicle groups 24 h after TBI (Figures 2A,B). However, the number of TUNEL-positive neurons was reduced after treatment with SIN. To investigate the effect of SIN on neuronal apoptosis, we have measured expression of the apoptosis-related proteins Bcl-2 and the level of cleaved caspase-3. Bcl-2 is a major anti-apoptotic member of the Bcl-2 family, and protects cells against a variety of insults such as exposure to calcium ionophores, glutamate, free radicals and withdrawal of trophic factors (Reed, 1998; Strauss et al., 2004). Cleaved caspase-3 is an essential component of the apoptotic machinery in many cell types (Yuan and Yankner, 2000; Engel et al., 2011).

Bcl-2 expression was lower in the TBI and TBI + vehicle groups than in the sham group 24 h after TBI, but elevated after SIN administration (Figure 2C). Content of cleaved caspase-3 was elevated in the TBI and TBI + vehicle groups and reduced after treatment with SIN (Figure 2D). These data demonstrate that SIN successfully inhibited neuronal apoptosis induced by TBI.

### SIN attenuates oxidative stress caused by TBI

**Figure 3:**
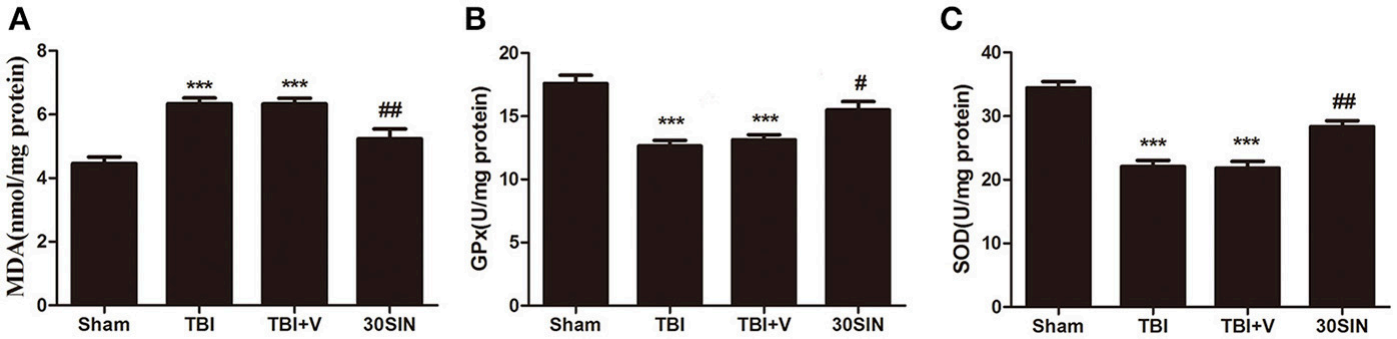
**SIN reduces oxidative stress in brain tissue following TBI. (A–C)** Oxidative stress was evaluated by the level of malondialdehyde (MDA) and the activities of glutathione peroxidase (GPx) and superoxide dismutase (SOD). Mice were subjected to TBI and then treated with SIN (30 mg/kg) or vehicle 30 min after TBI. As shown, **(A)** the level of MDA in the cortex increased remarkably 24 h after TBI. SIN treatment significantly suppressed the production of MDA after TBI. **(B,C)** The activities of GPx and SOD were decreased after TBI, while SIN treatment increased the activities of GPx and SOD. *n* = 6 per group. Data are presented as mean ± SEM. ^***^P < 0.001 vs. sham group, ^#^*P* < 0.05, ^##^*P* < 0.01 vs. TBI + vehicle group.

To evaluate whether the neuroprotective effect of SIN was derived from its ability to reduce TBI-induced oxidative stress, levels of MDA, GPx and SOD, indicators of lipid peroxidation and antioxidant enzyme activity, were measured in brain tissue. The TBI + vehicle group had a higher level of MDA than the sham group (*P* < 0.001) (Figure 3A), and there was no difference between the TBI and TBI + vehicle groups (*P* > 0.05). However, the level of cortical MDA in mice treated with SIN was significantly lower than in the TBI + vehicle group (*P* < 0.001) (Figure 3A). Activity of GPx and SOD, antioxidant enzymes responsible for scavenging metabolites generated by free radicals, was significantly lower after TBI than after sham injury (both *P* < 0.001), whereas the SIN-treated group showed significant upregulation of GPx and SOD activity (*P* < 0.05 and *P* < 0.01, respectively) (Figures 3B,C).

### SIN markedly promotes translocation of Nrf2 from cytoplasm to the nucleus in the cortex at 24 h after TBI

**Figure 4:**
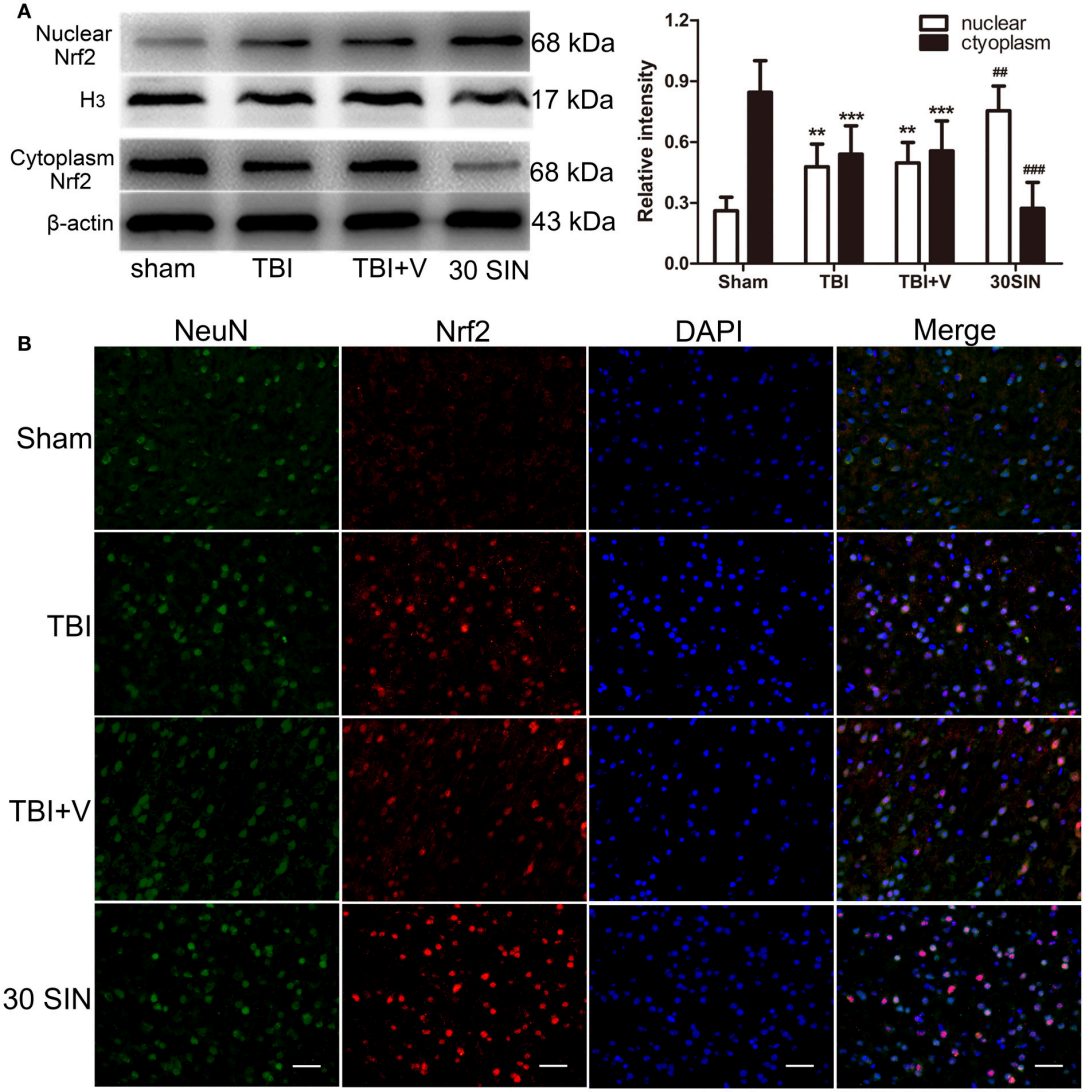
**SIN promoted translocation of Nrf2 from cytoplasm to the nucleus. (A)** Mice brain tissues were collected 1 day after TBI in different groups and the Nrf2 levels in both cytoplasm and nucleus were measured by Western blot. SIN significantly increased the level of Nrf2 in the nucleus and decreased the level of Nrf2 in the cytoplasm. **(B)** Representative immunofluorescence staining of Nrf2 after SIN treatment in mice with TBI. Double immunofluorescence analysis was performed with Nrf2 antibodies (red) and neuronal marker (green), and nuclei were fluorescently labeled with DAPI (blue). Scale bar = 20 μm. *n* = 6 per group. Data are presented as mean ± SEM. ^**^P < 0.01 and ^***^P < 0.001 vs. sham group; ^##^*P* < 0.01 and ^###^*P* < 0.001 vs. TBI + vehicle group.

The data obtained demonstrated that SIN has significantly reversed the parameters of oxidative stress induced by TBI (Figure 3). Because Nrf2 is a key protein in the reduction of oxidative stress, it was reasonable to hypothesize that SIN might activate Nrf2, thereby enhancing the activity of the antioxidant enzymes. Compared with the sham-injured group, both TBI and SIN administration induced Nrf2 nuclear translocation (Figure 4A). In addition, the SIN-treated group showed significantly greater nuclear expression of Nrf2, and lower cytoplasmic expression, than the TBI group that received vehicle (*P* < 0.01 and *P* < 0.001, respectively; Figure 4A), which demonstrated that Nrf2 was activated and SIN promoted Nrf2 nuclear translocation.

This effect was confirmed by immunofluorescence. Nrf2 expression in the sham-injured group was weak compared with the TBI or vehicle-treated groups and was predominately detected in the neuronal cytoplasm. Compared with the sham-injured group, there were more Nrf2-immunoreactive neurons in the TBI and vehicle-treated groups, with some translocation of Nrf2 from the cytoplasm to the nucleus being observed. In the SIN-treated group, there was more Nrf2-immunoreactivity detected in cell nuclei 24 h after TBI than in the vehicle group (Figure 4B). Together, this provides abundant evidence that SIN promotes Nrf2 translocation from the cytoplasm to the nucleus, thus improving its ability to bind to downstream genes.

### SIN upregulates the expression of Nrf2 downstream factors

**Figure 5:**
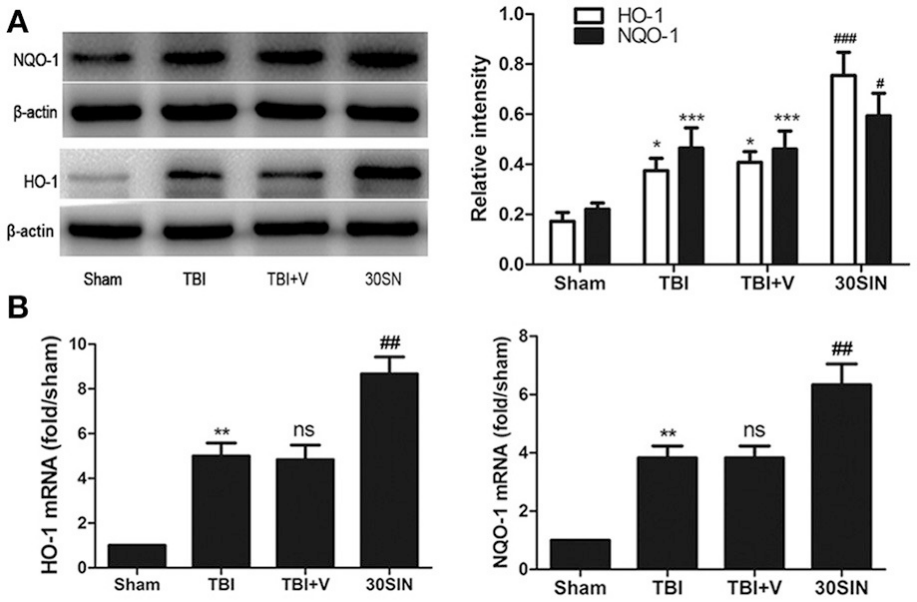
**SIN upregulated the expression of Nrf2 downstream factors in mice at both protein and mRNA levels**. Mice were subjected to TBI and treated with vehicle or SIN (30 mg/kg) 30 min after TBI. Protein and mRNA expression was measured by Western blot and RT-PCR, respectively. **(A)** Both HO-1 and NQO-1 proteins were upregulated after TBI; additionally, SIN further increased their expression in brain tissue. Actin was used as a loading control. Relative intensity means NQO-1 or HO-1/β-actin. **(B)** NQO-1and HO-1 mRNA were elevated after TBI and were further increased with administration of SIN. *n* = 6 per group. Data are presented as mean ± SEM. ^*^P < 0.05, ^**^P < 0.01, and ^***^P < 0.001 vs. sham group; ^ns^*P* > 0.05 vs. TBI group; ^#^*P* < 0.05, ^##^*P* < 0.01 and ^###^*P* < 0.001 vs. TBI + vehicle group.

Because SIN was able to activate Nrf2 and provide neuroprotection against TBI, we hypothesized that it might also regulate downstream factors in the Nrf2 pathway. We therefore measured the expression of NQO-1 and HO-1. At the protein level, NQO-1 and HO-1 were both upregulated after TBI (*P* < 0.001 and *P* < 0.05, respectively; Figure 5A). Additionally, administration of SIN further enhanced protein expression compared with vehicle (*P* < 0.05 and *P* < 0.001, respectively; Figure 5A). At the mRNA level, consistent with the protein changes, SIN enhanced the expression of NQO-1 and HO-1 compared with vehicle (both *P* < 0.01; Figure 5B). These results demonstrate that SIN induced the expression of factors downstream of Nrf2 in terms of protein and mRNA levels, via activation of the Nrf2 and the Antioxidant Responsive Element (Nrf2-ARE) signaling pathway.

## Discussion

In the present study, we evaluated the neuroprotective effects of SIN on TBI-induced brain injury in a mouse model and the underlying molecular mechanisms. The main findings of this study are as follows. (1) Administration of SIN improved neurological function, alleviated cerebral edema, and inhibited neuronal apoptosis. (2) SIN mitigated TBI-induced oxidative stress, which is represented by the level of MDA and the activity of GPx and SOD. (3) The translocation of Nrf2 from the cytoplasm to the nucleus after TBI, and SIN further promoted this translocation, subsequently increased the expression of downstream factors on mRNA and protein levels. These results suggest that SIN provides neuroprotection in a mouse model of TBI via the Nrf2-ARE pathway.

Secondary brain injury after TBI represents consecutive pathological processes involving oxidative stress, glutamate excitotoxicity, loss of ionic homeostasis, inflammatory response, and increased vascular permeability (Werner and Engelhard, 2007; Bell et al., 2009; Cornelius et al., 2013). Accumulating evidence shows that oxidative stress is a crucial contributor to the pathophysiology of TBI and mediates subsequent histopathology and neurobehavioral deficits (Bains and Hall, 2012). The enhanced production of reactive oxygen and nitrogen species though several different cellular pathways plays a pivotal role in TBI (Lewén et al., 2000; Cornelius et al., 2013; Radak et al., 2013). Moreover, these radicals can lead to lipid peroxidation, protein nitration and oxidation (Ansari et al., 2006), and DNA damage (Shao et al., 2006; Ansari et al., 2008). Oxidative stress may be a novel therapeutic target in TBI. MDA levels reflect lipid peroxidation (Hou et al., 2012; Cornelius et al., 2013), and begin to increase immediately after TBI, remaining elevated 48 h after injury (Hou et al., 2012). SOD and GPx are antioxidant enzymes responsible for scavenging metabolites generated by free radicals, catalyzing the conversion of peroxides into nontoxic forms (Miller et al., 2009; Cornelius et al., 2013). GPx is an intracellular antioxidant enzyme, converting peroxides into nontoxic forms (Miller et al., 2009; Cornelius et al., 2013). In the present study, SIN treatment after TBI reduced MDA level and increased GPx and SOD activity, suggesting that SIN could attenuate TBI-induced oxidative stress.

Increasing evidence indicates that Nrf2 is indispensable in the induction of antioxidant enzymes, as revealed in various CNS conditions (Wang et al., 2007; Yan et al., 2009; Chen et al., 2011) and Nrf2 and phase II enzymes, such as NQO1 and HO-1, were activated after TBI (Yan et al., 2008, 2009). Besides, Nrf2-/- mice exhibited poorer outcomes than the wild-type mice, while activation of Nrf2 could protect against brain injury after TBI (Wang et al., 2012). Our results also showed that Nrf2 translocated from the cytoplasm to the nucleus after TBI. These evidences indicate that activation of the Nrf2–ARE pathway is beneficial for TBI.

SIN has been shown to exert neuroprotection in several CNS disease models. Zhao and colleagues (Yang et al., 2014) reported that SIN attenuates brain injury from intracerebral hemorrhage by inhibiting activation of microglia. Wu et al. (2011) demonstrated that SIN is potently neuroprotective against ischemic brain injury when administered before or after the injury. In a recent study, Qin et al. (2016) showed that SIN upregulates Nrf2 and phase-II enzymes to exert renoprotective effects in mouse fibrotic kidney. We therefore hypothesized that SIN could also activate the Nrf2-ARE pathway in the brain after TBI to alleviate brain injury by inhibiting oxidative stress secondary to the upregulation of antioxidant enzymes. In the present study, we investigated the changes in the Nrf2-ARE signaling pathway after SIN administration. The data showed that Nrf2 translocated from the cytoplasm to the nucleus after TBI, and SIN further promoted this translocation. Compared with the TBI and TBI + vehicle groups, SIN administration also significantly upregulated the expression of HO-1 and NQO1 at the pre- and post-transcriptional levels 24 h after TBI. HO-1 and NQO1 are potent antioxidant and detoxifying enzymes. HO-1 protects against a number of pathophysiological insults including oxidative stress (Miller et al., 2009). NQO1 is able to protect cells against the adverse effects of quinones and related compounds (Radjendirane et al., 1998). Accordingly, SIN administration reduced the oxidant damage and alleviated brain injury in the TBI models in this study. However, the mechanism of activation of the Nrf2–ARE pathway by SIN has not been completely elucidated. A number of kinases, such as phosphoinositol-3 kinase and extracellular signal-regulated protein kinase reportedly phosphorylate Nrf2 directly and affect its cellular location or stability in response to some stimuli (Zipper and Mulcahy, 2003; Tsai et al., 2013). Furthermore, protein kinase C (PKC) could disrupt the interaction between Nrf2 and KEAP1 by directing phosphorylation of Nrf2 (Huang et al., 2000). These mechanisms may be involved in the activation of the Nrf2–ARE pathway by SIN after TBI, and need to be investigated in future studies.

Our study has several limitations. First, SIN was only administered once; we do not know whether multiple treatments with different time courses would be as effective. Regarding the clinic application, the therapeutic window of 30 min which was employed in this study is narrow. Thus, other time points later than 30 min post-TBI should be tested. Second, SIN may have other protective effects against TBI that were not evaluated in this study, such as anti-inflammatory and immunomodulatory properties. Lastly, we did not use Nrf2 gene knockout mice, which would confirm whether the observed effects of SIN are due to activation of the Nrf2–ARE pathway. Further comprehensive studies are warranted.

## Conclusion

To the best of our knowledge, the present study is the first to demonstrate the effects of SIN on the Nrf2–ARE signaling pathway in a model of TBI. Our data show that SIN treatment 30 min after TBI ameliorates secondary brain injury by improving neurologic function, reducing brain edema, combating oxidative stress and attenuating neuronal apoptosis. These effects might correlated with translocation of Nrf2 from the cytoplasm to the nucleus and activation of downstream proteins.

## Author contributions

YY: Designed the study, performed the TBI model and biochemical analysis and wrote the manuscript. LL and XL: Prepared the drug solutions and performed histological examination. QW, HD, and XW: Performed the TUNEL staining and the animal studies. ZY, LW, and XZ: Contributed to the Western blotting. MZ and HP: Designed the animal studies. HW: Contributed to the design and analysis of the study and wrote the manuscript. All authors approved the final version of the manuscript.

### Conflict of interest statement

The authors declare that the research was conducted in the absence of any commercial or financial relationships that could be construed as a potential conflict of interest.

## Abbreviations

TBI: traumatic brain injury
Nrf2: nuclear factor erythroid 2-related factor 2
Keap1: Kelch-like ECH-associated protein 1
SIN: sinomenine
NQO1: NAD(P)H:quinone oxidoreductase 1
HO1: heme oxygenase 1
MDA: malondialdehyde
GPx: glutathione peroxidase
SOD: superoxide dismutase
ROS: reactive oxygen species
RT-PCR: real-time quantitative polymerase chain reaction
TUNEL: terminal deoxynucleotidyl transferase-mediated dUTP nick end labeling
Bcl-2: B-cell lymphoma 2
CNS: central nervous system
SDS-PAGE: sodium dodecyl sulfate-polyacrylamide gel electrophoresis
PBS: phosphate buffer solution
PVDF: polyvinylidene fluoride
DAPI: 4′,6-diamidino-2-phenylindole
i.p.: intraperitoneally.
